# Organ Metallome Processed with Chemometric Methods Enable the Determination of Elements that May Serve as Markers of Exposure to Iron Oxide Nanoparticles in Male Rats

**DOI:** 10.1007/s12011-020-02104-z

**Published:** 2020-03-12

**Authors:** Marzena Rugiel, Agnieszka Drozdz, Katarzyna Matusiak, Zuzanna Setkowicz, Krzysztof Klodowski, Joanna Chwiej

**Affiliations:** 1 Faculty of Physics and Applied Computer Science, AGH University of Science and Technology, Krakow, Poland; 2 Jagiellonian University, Institute of Zoology and Biomedical Research, Krakow, Poland

**Keywords:** Iron oxide nanoparticles, Elemental analysis, Total reflection X-ray fluorescence microscopy, Multivariate methods, Cluster and discriminant analysis

## Abstract

The systemic influence of iron oxide nanoparticles on the elemental homeostasis of key organs was examined in male rats. In tissues taken at different intervals from nanoparticles injection, the dynamics of elemental changes was analyzed. The organ metallome was studied using total reflection X-ray fluorescence. The obtained data were processed with advanced cluster and discriminant analyses—to classify the tissues according to their organs of origin and to distinguish accurately the nanoparticle-treated and normal rats. Additionally, in the case of liver and heart, it was possible to determine the elements of highest significance for different treatments, which may serve as markers of exposure to iron oxide nanoparticles.

**Electronic supplementary material:**

The online version of this article (10.1007/s12011-020-02104-z) contains supplementary material, which is available to authorized users.

## Introduction

Nanoparticles (NPs) are defined as particles with two or three dimensions in the range from 1 to 100 nm. Their properties depend on the shape, chemical composition, and size which are comparable to biological molecules and subcellular structures [1, 2]. Among different nanomaterials, iron oxide nanoparticles (IONPs) have the greatest potential to serve for biomedical purposes. Because of their magnetic properties, IONPs can be used as contrast agents in magnetic resonance imaging, and their ability to interact with the external magnetic field gradients makes them potentially ideal drug carriers [2–6]. Furthermore, in response to an external magnetic field, they can also induce local hyperthermia in pathologically changed tissues [2, 4]. Thus, the use of IONPs may improve effectiveness of medical diagnostics and therapy and/or reduce their negative side effects [2, 4, 6].

For successful development of IONPs medical application, it is necessary to monitor their potential toxicity considering relationships between the shape, size, or chemical properties of the surface of the nanomaterials including their in vivo behavior [1, 7, 8]. It is important to verify the final fate of NP-based drug carriers after the drug release at the site of action and to determine whether and how the nanomaterials themselves are metabolized, degraded, and/or effectively removed from the body [1, 3].

The essential stage of the in vivo investigations is animal tests, which allow to follow the systemic impact of NPs on a living organism [9, 10]. The evidence gained through such a study points out that IONPs may accumulate within organs such as liver, brain, heart, or kidneys, leading to transient histopathological and functional changes [11–17]. One of the possible ways to obtain information about the biodistribution and biokinetics of IONPs as well as the range of their effects is exploration of elemental changes occurring in organs of NPs-treated animals [9, 18–22]. In combination with multivariate statistical analyses, this approach may allow to determine elemental markers of biological effects following exposure to nanoparticles.

Multivariate methods improve the analysis of multidimensional data, allowing useful comparisons to be made from the elemental composition of the samples [23, 24]. The most important ones for consideration here include discriminant analysis and cluster analysis, the main goals of which are simplification of the data structure, grouping observations, and investigation of the relationship between variables [25–27].

In this work, the utility of multivariate methods for the determination of the elemental markers of exposure to low doses of polyethylene glycol–coated IONPs (PEG-IONPs) was investigated. For this purpose the main organs taken from three groups of rats treated with PEG-IONPs and normal animals were examined. Three different time intervals counting from NPs administration were studied, namely 2 h, 24 h, and 7 days from the injection. Cluster analysis was used for unsupervised classification of organs and examined groups of animals. Similar two approaches were used in the case of discriminant analysis. However, the latter classification was conducted into the populations defined a priori, before analysis. Additional purpose of the discriminant analysis was determination of the original variables (elements) playing the greatest role in the process of groups discrimination.

## Materials and Methods

### Experimental Animals and Sample Preparation

The tissues subjected to elemental analysis were obtained from male Wistar rats, which came from the colony of the Department of Neuroanatomy, Institute of Zoology and Biomedical Research, Jagiellonian University in Krakow. All procedures related to the use of animals were carried out according to the agreement no. 121/2015 of the First Local Ethical Committee on Animal Testing at the Jagiellonian University in Krakow and were performed in accordance with the international standards. The animals were grown at the constant temperature (20 ± 2 °C), with unrestricted access to food (Labofeed) and maintaining 12 h/12 h light/dark cycles. On the 60th day of their postnatal development, 24 rats were divided into four equinumerous groups and three of them were injected through the tail vein with 1 ml of PEG-IONPs solution (described below), while the controls (group N, *n* = 6) were treated with the same volume of saline.

PEG-coated magnetic iron (II, III) oxide nanoparticles (Sigma-Aldrich 747,408) were dispersed in 15% mannitol (Baxter), and the final concentration of Fe in the injected solution was 8.14 ppm. The hydrodynamic diameter and zeta potential of NPs (both values determined in mannitol solution after removing of the aggregates/agglomerates) were equal to 35 nm and − 98 mV, respectively.

Three groups of NPs-treated animals were anesthetized and perfused with saline 2 h (2H group, *n* = 5^1^), 24 h (24H group, *n* = 6) and 7 days (7D group, *n* = 6) from the injection. The selected organs (brain, heart, kidneys, liver, spleen, and muscle) were taken from the animal body immediately after the perfusion. The elemental analysis was done on the liquid organ samples which were obtained in the process of digestion in high purity 65% nitric acid. This was carried out in the Speedwave 4 microwave digestion system (BERGHOF). As an internal standard, gallium solution in concentration of 10 ppm was used at 0.3 ml per 1 ml of digested tissue sample. A detailed protocol of the whole procedure can be found elsewhere [20, 22].

In the in vivo studies of IONPs toxicity that have been carried out so far, animals were exposed to relatively high doses of Fe. They equaled from 0.8 to even 12 mg Fe per kg of body mass and were equal or much higher than doses used clinically in humans (~mgs of Fe per kg of body weight) [28–31]. In our experiment, each animal obtained 0.00814 mg of Fe which may seem little. However, if we do simple calculations taking into account (1) the typical Fe contents in wet mass of rat organs [32], (2) the masses of examined organs in adult rats, and (3) the mass of administered iron, we can easily show that the injected Fe constitutes from around 1% (for the liver) to even 35% (for the kidney) of total Fe mass in the organ and, the same, might affect the elemental homeostasis of the rat organism. In the context, we need also to take into account the route of administration, as in our study, NPs were injected directly to blood and not administered orally or intraperitoneally.

### Elemental Analysis

The elemental analysis of tissue samples was performed using the total reflection X-ray fluorescence (TXRF) method in the Laboratory of X-ray Methods of the Institute of Physics at the Jan Kochanowski University in Kielce. S2 PICOFOX (Bruker Nano) automatic analytical instrument was used for this purpose. The details concerning the used apparatus and measurement conditions were shown in our previous papers [20, 22].

The analysis of TXRF spectra allowed a quantitative analysis of the following elements in the measured tissues: P, S, K, Ca, Fe, Cu, Zn, and Se. Their concentrations in the liver, heart, brain, spleen, kidneys, and muscles in each of the examined animal were the subject of further statistical study including those using multivariate methods.

The calculations necessary to obtain information about the concentrations of elements present in the samples were done in two steps. First, the concentration *C*_*i*_ of the element *i* in the measured liquid sample was calculated according to Formula (1). In turn, the final elemental concentration ${C}_i^x$ within organ was calculated, taking into account dilution factor *d* and the liquid to organ mass conversion factor *f*_*x*_ what was described by Eq. (2).

1 $$ {C}_i=\frac{C_{IS}\cdotp {N}_i}{N_{IS}\cdotp {s}_i} $$

where:

*C*_*IS*_−: concentration of the internal standard (Ga) in the liquid sample (ppm),
*N*_*IS*_−: net pulse number for the internal standard in the sample spectrum (a.u.),
*N*_*i*_−: net pulse number for the element *i* in the sample spectrum (a.u.),
*s*_*i*_−: relative sensitivity for the element *i*.

2 $$ {C}_i^x={C}_i\cdotp d\cdotp {f}_x $$

where:

$$ {C}_i^x- $$
the elemental concentration in the organ *x* (ppm),
*C*_*i*_−: the elemental concentration in the diluted sample (ppm),
*d*−: dilution coefficient (a.u.),
*f*_*x*_−: liquid to organ mass conversion factor for organ *x* (a.u.).

### Multivariate Statistical Analysis

Two multivariate methods, namely cluster and discriminant analysis, were used for advanced statistical evaluation of the elemental data.

Cluster analysis is typically used to search for patterns in a data set by grouping the observations into clusters in such a way that objects within each cluster are similar while the clusters are dissimilar to each other. Two approaches are used for this purpose—hierarchical and divisive methods. In the case of hierarchical clustering, in the beginning, each observation is treated as a single cluster. Further combining of observations into clusters (agglomeration) occurs with the increase of the degree of non-similarity of objects until one cluster containing all observations is received. In divisive methods, this process is reversed, but in both cases, the result is a structure called a dendrogram [25].

There are several hierarchical methods of clustering (nearest neighbor, farthest neighbor, weighted pair-group average method, etc.); however, the most popular is Ward’s method using an analysis of variance approach to create the links. The sums of squares of deviations within the clusters are minimized, and at each level of combining of clusters, a pair creating the cluster with the least possible diversity is chosen [25]. Ward’s method was applied also in this work.

Grouping of objects into clusters is possible, thanks to the dissimilarity function being the measure of the distance between the observations. The most commonly used distance measures are the Euclidean or squared Euclidean distances. The Euclidean distance between the two vectors *x* = (*x*_1_, *x*_2_, …, *x*_*p*_) and *y* = (*y*_1_, *y*_2_, …, *y*_*p*_) is defined as [25]:

3 $$ d\ \left(x,y\right)=\sqrt{\sum_{j=1}^p{\left({x}_j-{y}_j\right)}^2} $$

The main task of the discriminant analysis is to confirm the existence of differences between two or more groups to which the objects were assigned. To elucidate these differences, linear functions of the variables (discriminant functions) are used. Discriminant functions are determined in such a way that the external variability (between populations) is significantly greater than the internal variability (between the objects within the same populations). In order to check which discriminant functions are necessary to differentiate groups, Wilks’ *Λ* statistics, which can be approximated with a chi-square distribution, is used. In turn, *F* Fisher’ statistics corresponds to partial Wilks’ *Λ*, which is utilized to determine the variables playing the greatest role in the process of discrimination [25].

The choice of methods of multivariate analysis in this paper was motivated by the nature of the samples tested. In our study, each object was described by a set of features which constituted the concentrations of particular elements.

It is necessary to mention that the data set used within this study has been partially utilized before in the papers of Matusiak et al. [22] and Skoczen et al. [20]. In the study of Matusiak et al., the changes in the contents of 4 elements, namely Ca, Fe, Cu, and Zn, were examined only in the liver of animals exposed to NPs action and controls, while in the work of Skoczen et al., the anomalies of the same 4 elements were measured for the remaining organs (kidneys, spleen, heart, and brain). In this paper, for the first time, we examined the whole data set obtained during the animal experiment, which now include also information about changes occurring in the accumulation of P, S, K, and Se. Besides the analysis of additional elements and tissue (muscle), the obtained results were based on a different research approach in which all the data were treated as one set of variables. The used solution was justified by the European Union directive on the use of laboratory animals, which obliges researchers to comply with the 3Rs principle. One of its key elements is the reduction of the number of animals while maintaining the statistical significance of subsequent analyses. In our work, this principle was respected, trying to use all information from the collected till now material. Repetition of the experiment on new animals in order to obtain a relatively small, complementary material that can be obtained from the same samples would be inconsistent with the recommended research standards.

## Results

The result of the quantitative elemental analysis were the concentrations of elements (P, S, K, Ca, Fe, Cu, Zn, and Se) in the main internal organs (liver, heart, brain, spleen, kidneys, and muscles marked as Li, He, Br, Sp, Ki, Mu, respectively). As the first step, the median concentrations of elements were calculated and the differences between PEG-IONPs-treated animals and controls were tested using non-a parametric *U* Mann-Whitney test at the 5% level of statistical significance. The obtained median values together with their uncertainties calculated as interquartile spans are presented in Table 1. The statistically significant differences between rats injected with NPs and normal animals were also marked there.

**Table 1:** Concentrations of elements in the organs of examined rat groups*

Group	P		S		K		Ca		Fe		Cu		Zn		Se	
	Median (ppm)	IQR** (ppm)	Median (ppm)	IQR (ppm)	Median (ppm)	IQR (ppm)	Median (ppm)	IQR (ppm)	Median (ppm)	IQR (ppm)	Median (ppm)	IQR (ppm)	Median (ppm)	IQR (ppm)	Median (ppm)	IQR (ppm)
Liver																
N	5754	921	2978	198	5442	682	1299	818	137	5	8.37	0.83	125	38	1.249	0.134
2H	5847	392	2950	275	5318	231	1912	1549	154	27	*7.14*	*0.70*	161	74	1.245	0.066
24H	5336	359	2827	93	*4629*	*210*	1771	367	118	29	*6.22*	*0.73*	134	21	*1.154*	*0.086*
7D	5046	587	*2706*	*221*	*4564*	*262*	*2984*	*681*	119	62	*5.95*	*0.32*	*206*	*38*	*1.116*	*0.158*
Spleen																
N	5459	1053	2366	561	5922	1286	2072	7737	547	239	1.90	0.17	159	482	0.521	0.026
2H	5038	592	2149	421	5603	945	3410	5083	461	137	1.86	0.26	119	318	0.393	0.037
24H	5151	669	2378	385	5797	847	4539	5922	462	69	1.92	0.36	248	323	0.482	0.091
7D	5409	557	2324	431	5708	427	5413	8570	510	40	1.79	0.28	304	381	0.452	0.078
Kidney																
N	4233	302	2291	183	2922	235	121	278	45	7	6.37	0.90	32	17	1.642	0.176
2H	4940	802	*2738*	*349*	*3629*	*507*	*2257*	*1191*	*55*	*9*	7.17	0.51	*170*	*87*	1.760	0.213
24H	4571	568	2440	536	3325	732	139	4587	45	7	*8.08*	*0.98*	33	268	1.882	0.372
7D-Ki	*4749*	*406*	2687	284	*3575*	*431*	*1783*	*2279*	*53*	*10*	*7.95*	*1.42*	*142*	*154*	1.899	0.220
Brain																
N	6114	518	2028	213	6095	655	732	2001	21	6	3.78	0.81	50	141	0.164	0.044
2H	*5316*	*557*	1783	210	5740	425	494	575	18	3	3.44	0.10	45	43	*0.135*	*0.012*
24H	5736	439	2020	198	5896	217	602	318	19	3	3.52	0.16	41	6	0.165	0.040
7D	*5141*	*426*	1829	227	*5561*	*378*	585	768	19	4	3.46	0.59	40	14	0.144	0.008
Muscle																
N	4265	124	3255	197	6114	509	1525	4183	30	5	2.35	0.16	58	240	0.124	0.025
2H	3834	125	2994	255	5682	228	*748*	*548*	*24*	*1*	2.08	0.20	*34*	*17*	0.086	0.130
24H	4143	316	3156	141	6009	295	847	1177	25	7	2.12	0.32	35	42	0.102	0.044
7D	4014	427	3143	369	5832	113	2665	3716	27	5	2.04	0.39	103	87	0.124	0.007
Heart																
N	2966	318	2493	335	3471	244	112	411	75	16	6.61	0.28	25	6	0.364	0.040
2H	3159	257	2535	117	3746	51	900	1762	77	12	6.49	0.11	*82*	*149*	*0.295*	*0.030*
24H	*3329*	*324*	*2752*	*173*	*3845*	*316*	832	2606	82	7	6.84	0.83	72	116	0.356	0.031
7D	3043	46	2491	179	*3646*	*376*	1214	287	80	4	6.81	0.33	*93*	*31*	0.355	0.066
Detection limits for normal animals																
Liver	19.5	4.3	5.15	0.87	2.37	0.51	1.49	0.44	0.24	0.16	0.095	0.018	0.092	0.018	0.0413	0.0051
Spleen	20.5	4.8	7.04	0.72	3.14	0.63	1.55	0.86	0.234	0.042	0.12	0.029	0.129	0.032	0.0540	0.0063
Kidney	13.4	3.5	5.5	1.7	1.79	0.57	0.68	0.23	0.123	0.04	0.076	0.025	0.078	0.027	0.042	0.013
Brain	13.1	1.9	4.80	0.65	2.11	0.35	0.83	0.32	0.114	0.018	0.076	0.012	0.074	0.011	0.0386	0.0071
Muscle	29.5	9.9	10.4	3.4	4.6	1.7	2.1	1.5	0.27	0.11	0.18	0.077	0.181	0.072	0.088	0.035
Heart	11.0	2.1	4.56	0.64	1.64	0.32	0.61	0.28	0.105	0.016	0.063	0.012	0.062	0.016	0.0319	0.0037

*Statistically significant differences in concentrations comparing with normal rats were in italics; The statistically significant anomalies should be marked in red or blue, depending on their values comparing to control group

**IQR, interquartile range

The only organs sharing statistically significant differences in Fe levels were the kidneys (Table 1). As a result of PEG-IONPs injection, the level of Fe increased in kidneys after 2 h and 7 days from the injection. Nonetheless, the treatment of animals with NPs, led to many other significant elemental abnormalities of the examined organs, which were described and discussed in details in our previous papers [20, 22].

In this paper, the data on accumulation of elements in organs have been treated as a set of features describing each animal (object) and, for the purpose of their simultaneous comparisons, two multivariate methods (cluster and discriminant analysis) were applied. They were used in two approaches, for classification based on the examined organs and classification due to the analyzed groups of animals. The data were explored using the STATISTICA software (version 7.1).

### Evaluation of the Statistical Significance of Differences in the Elemental Composition of Main Organs

The first approach allowed to check whether samples of the examined organs can be distinguished based on their elemental composition. The analyses were carried out for each animal group (N, 2H, 24H, 7D) separately.

#### Cluster Analysis

**Fig. 1:**
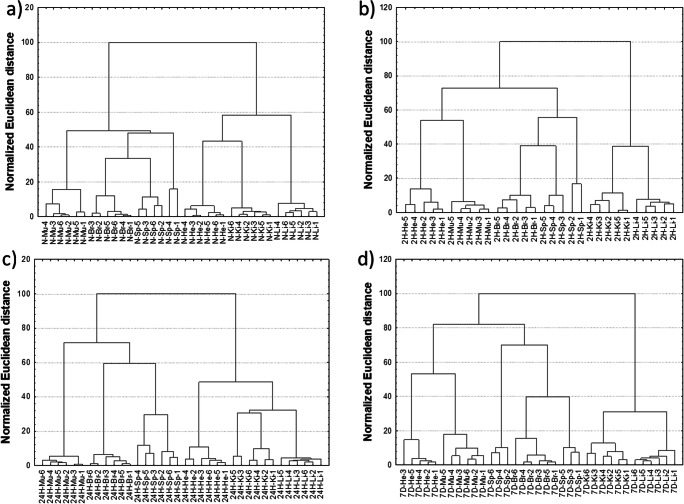
Dendrograms depicting the results of unsupervised classification of organs for normal rats (**a**) and animals treated with IONPs during 2 h (**b**), 24 h (**c**), and 7 day (**d**) periods from injection. **d** Number of heart samples in 7D group was equal to 5, because one organ was lost during the sample preparation procedures.

Cluster analysis was performed on the concentrations of elements after the data standardization using the STATISTICA software. The hierarchical Ward method of agglomeration and the Euclidean distance as a measure of the distance between the observations were applied. The results are presented in the form of dendrograms in Fig. 1. For all the studied animal groups, the samples from particular organs form distinct clusters. However, in the case of spleen, the allocation of all samples into one cluster was observed only in the 24H group, while for the N, 2H and 7D groups, some of the spleen samples showed a greater similarity to the samples originating from the brain. In addition, the shapes of dendrograms for animals from the N and 24H groups as well as the 2H and 7D groups presented pairwise similarities. In the case of the first pair, two distinct clusters could be observed. The first one included muscle, brain, and spleen, while the second one—heart, kidney, and liver samples. For the 2H and 7D groups, three distinct clusters could be noticed instead of two. The first of them included samples from muscle and heart, indicating similarity of their elemental composition. The second cluster consisted of brain and spleen samples, while the third contained kidney and liver tissues. It is worth to mention that the classification of heart samples together with muscle samples is the main differentiator between the second (2H and 7D) and the first pair of groups (N and 24H).

#### Discriminant Analysis

First, the analysis was done for the N group using a standard method in which all primary variables were taken into account. Five discriminant functions were distinguished and their characteristics are presented in Table 2.

**Table 2:** Characteristics of canonical discriminant functions obtained for the N group

Discriminant function	Eigenvalue	Canonical correlation	Wilks’ Λ	Chi-square statistics	Df*	*p* value**
1	209.6	0.998	0	463	40	< 0.05
2	61.8	0.992	0.000014	313	28	< 0.05
3	31.1	0.984	0.000853	197	18	< 0.05
4	8.7	0.947	0.027378	100	10	< 0.05
5	2.8	0.857	0.264784	37	4	< 0.05

*Df—number of degrees of freedom for chi-square statistics

**p value—level of statistical significance

The data presented in Table 2 confirmed the statistical significance of all the obtained discriminant functions. Therefore, their raw canonical coefficients are shown in Table 3.

**Table 3:** Raw canonical coefficients of discriminant functions determined for the N group

Element	Discriminant function				
	1	2	3	4	5
Constant value	− 4.3266	− 0.8795	0.3390	− 3.8913	11.4237
P	0.0021	0.0036	0.0025	− 0.0009	0
S	0.0013	− 0.0023	− 0.0065	0.0020	− 0.0012
K	− 0.0041	− 0.0009	− 0.0006	− 0.0001	− 0.0013
Ca	− 0.0001	− 0.0003	− 0.0006	− 0.0014	0.0001
Fe	− 0.0045	0.0128	0.0078	0.0152	0.0013
Cu	0.0601	− 2.3282	2.3391	0.6024	− 0.3032
Zn	0.0036	− 0.0005	0.0097	0.0216	− 0.0020
Se	16.5732	6.6111	− 7.9734	− 2.1838	− 0.9165

Based on the data from Table 3, five significant discriminant functions could be calculated and each object could be characterized by them instead of eight original variables. The dependences describing how the first two discriminant functions depend on raw primary variables were presented in Eqs. (4) and (5). As one can see it, the discriminant functions are sums of the original variables weighted by respective raw coefficients of discriminant function.

4 $$ {D}_1=-4.3266+0.0021P+0.0013S\hbox{--} 0.0041K\hbox{--} 0.0001 Ca\hbox{--} 0.0045 Fe+0.0601 Cu+0.0036 Zn+16.5732 Se $$

5 $$ {D}_2=-0.8795+0.0036P-0.0023S\hbox{--} 0.0009K\hbox{--} 0.0003 Ca+0.0128 Fe-2.3282 Cu-0.0005 Zn+6.6111 Se $$

**Fig. 2:**
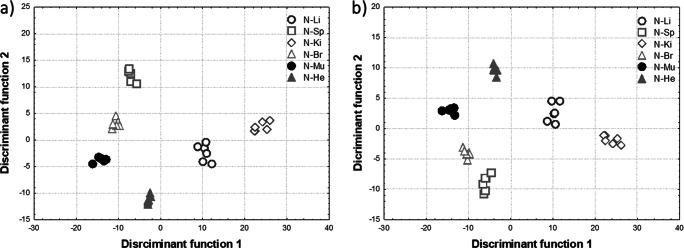
Scatterplots of observations in the space of discriminant variables obtained for the N group when (**a**) all the primary variables or (**b**) only the elements of highest significance for the model (P, S, K, Fe, Cu, and Se in the case of the N group) were included in the analysis

By replacing the primary variables with the *D*_1_ and *D*_2_ coordinates of the discriminant functions, a scatterplot of observations was obtained (Fig. 2a).

In order to determine the elements of the highest significance for the model, the partial Wilks lambda, *F* test of partial Wilks’ lambda, and its *p* value were used. All these parameters are shown in Table 4.

**Table 4:** Parameters describing the significance of the primary variables for the model (N group)

	Partial Wilks’ lambda	*F* Fisher’s statistics	*p* value
P	0.223	16.04	< 0.05
S	0.212	17.11	< 0.05
K	0.307	10.39	< 0.05
Ca	0.806	1.11	0.38
Fe	0.083	50.83	< 0.05
Cu	0.044	100.04	< 0.05
Zn	0.844	0.85	0.53
Se	0.032	138.69	< 0.05

Based on the data presented in Table 4, the elements of the greatest importance in the process of organ distinguishing in the case of the N group were determined. These were P, S, K, Fe, Cu, and Se; the elements for which the lowest partial Wilks lambdas, largest values of *F* Fisher statistics, and *p* values less than 0.05 were observed. Further analysis was carried out for these six elements, and the newly calculated raw canonical coefficients are presented in Table 5 and used to create a scatterplot of observations in the space of discriminant variables shown in Fig. 2b. Despite the reduced number of variables in the model, different organs taken from normal animals are still very well separated in the space of discriminant variables (Fig. 2b).

**Table 5:** Raw canonical coefficients of discriminant functions for the N group (only P, S, K, Fe, Cu, and Se are taken into account)

Element	Discriminant function				
	1	2	3	4	5
Constant value	− 4.6426	− 1.0535	− 0.1169	− 3.4667	− 11.665
P	0.0023	−0.0033	− 0.0025	− 0.0004	0
S	0.0012	0.0028	0.0064	0.0014	0.001
K	− 0.0041	0.0009	0.0006	− 0.0004	0.001
Fe	− 0.0030	− 0.0053	− 0.0069	0.0129	− 0.001
Cu	− 0.1407	1.9515	− 2.3879	0.5586	0.338
Se	17.0947	− 5.1035	7.8388	− 1.7549	0.853

A typically used measure of differences between the studied groups in the space of discriminant variables is the squared Mahalanobis distance between their centroids—meaning the centers of gravity of the observations in this space. The squared Mahalanobis distances between different organs representing the N group together with the *F* Fisher statistics and its *p* values are included in Table 6. All the *p* values presented there are less than 5%, which confirms the statistical significance of the calculated Mahalanobis distances and significance of the differences between the six analyzed organs for the N group.

**Table 6:** Squared Mahalanobis distances obtained for the N group

	Squared Mahalanobis distance						F- statistics						*p* value					
	Li	Sp	Ki	Br	Mu	He	Li	Sp	Ki	Br	Mu	He	Li	Sp	Ki	Br	Mu	He
Li	0	432	271	513	750	266	-	180	113	214	312	111	-	< 0.05	< 0.05	< 0.05	< 0.05	< 0.05
Sp	432	0	963	150	291	373	180	-	401	62	121	155	< 0.05	-	< 0.05	< 0.05	< 0.05	< 0.05
Ki	271	963	0	1281	1504	940	113	401	-	534	627	392	< 0.05	< 0.05	-	< 0.05	< 0.05	< 0.05
Br	513	150	1283	0	305	274	214	62	534	-	127	114	< 0.05	< 0.05	< 0.05	-	< 0.05	< 0.05
Mu	750	291	1504	305	0	303	312	121	627	127	-	126	< 0.05	< 0.05	< 0.05	< 0.05	-	< 0.05
He	266	373	940	274	303	0	111	155	392	114	126	-	< 0.05	< 0.05	< 0.05	< 0.05	< 0.05	-

An analogous approach was used for the 2H, 24H, and 7D groups. In all cases, it was shown that the examined organs were well separated in the space of discriminant variables. The elements contributing the most to the organ discrimination in the 2H group were S, K, Fe, Cu, Zn, and Se; in the 24H group P, S, Ca, Fe, Cu, and Se; and in the 7D group, P, S, K, Fe, Cu, and Se. Excluding of the redundant elements from the model allowed still good differentiation of examined organs in the space of discriminant variables what one can see from Figs. S1–S3 presented in the Supplementary materials. What is more, these qualitative results have been confirmed by statistics related to the squared Mahalanobis distances.

Discriminant analyses for N, 2H, 24H, and 7D groups are characterized in Table 7 by numbers of both all and statistically significant discriminant functions and the elements of the highest significance for the models determined based on partial Wilks’ lambdas.

**Table 7:** Summary of discriminant analysis results for groups N, 2H, 24H, and 7D

Group	Number of all discriminant functions	Number of significant discriminant functions	Elements significant for the model
N	5	5	P, S, K, Fe, Cu, Se
2H	5	5	S, K, Fe, Cu, Zn, Se
24H	5	5	P, S, Ca, Fe, Cu, Se
7D	5	4	P, S, K, Fe, Cu, Se

Accordingly to Table 7, all determined discriminant functions were statistically significant in the case of N, 2H, and 24H groups. Regardless of the studied animal population, the elements such as S, Fe, Cu, and Se played an important role in the process of organ differentiation. However, in order to obtain good separation of cases in the space of discriminant variables, depending on the studied group, one had to take into account P and K for N and 7D groups, K and Zn for 2H group, and P and Ca for 24H group, additionally.

### Evaluation of Differences in the Elemental Composition Between Organs from IONPs-Treated and Normal Rats

The second methodical approach allowed to check whether a tissue sample of given main organ could be assigned to a specific group of IONPs-treated animals or to untreated controls based on the analysis of its elemental composition.

#### Cluster Analysis

**Fig. 3:**
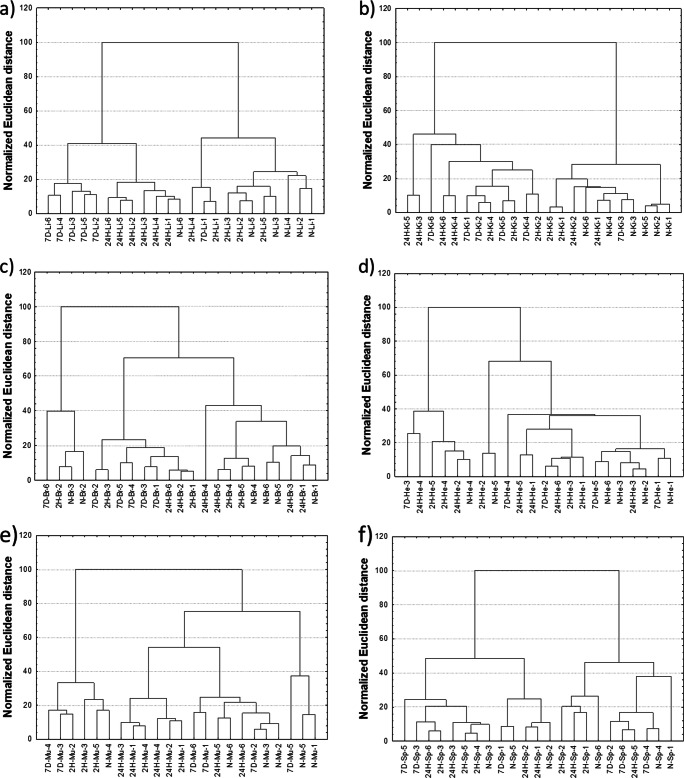
Dendrograms presenting the results of unsupervised classification of animals based on the elemental composition of the liver (**a**), kidneys (**b**), brain (**c**), heart (**d**), muscles (**e**), and spleen (**f**)

Cluster analysis was performed on the standardized concentrations of elements in the main internal organs. For this purpose, the Ward’s hierarchical method of agglomeration and the Euclidean distance as a measure of dissimilarity between objects were applied. The results obtained for each organs are presented in the form of dendrograms and compared in Fig. 3.

The cluster analysis carried out for the examined organs indicated that the differences in elemental composition of samples from the studied groups were too small to enable the separation of the population in the Ward agglomeration process. However, some regularity can be observed for the liver, for which the majority of cases representing four studied groups of animals were classified into separate clusters.

#### Discriminant Analysis

At the beginning, the analysis was done for the liver using a standard method in which all primary variables were taken into account. Three statistically significant discriminant functions were distinguished and their characteristics are presented in Table 8.

**Table 8:** Characteristics of canonical discriminant functions determined for the liver

Discriminant function	Eigenvalue	Canonical correlation	Wilks’ Λ	Chi-square statistics	Df*	*p* value**
1	4.0	0.895	0.019	64	24	< 0.05
2	3.1	0.870	0.094	38	14	< 0.05
3	1.6	0.784	0.386	15	6	< 0.05

*Df—number of degrees of freedom for chi-square statistics

**p value—observed significance level

As one can see it from Table 8, all separated discriminant functions were statistically significant. Therefore, for all of them, raw canonical coefficients are calculated and presented in the Table 9.

**Table 9:** Raw canonical coefficients of discriminant functions obtained for the liver

Element	Discriminant function		
	1	2	3
Constant value	− 11.0960	18.0359	− 1.1327
P	0.0017	− 0.0008	0.0028
S	0.0059	− 0.0026	0.0025
K	0	− 0.0005	− 0.0032
Ca	− 0.0083	0.0070	0.0014
Fe	0.0129	− 0.0032	− 0.0024
Cu	− 3.5453	0.6294	− 1.1682
Zn	0.1453	− 0.1160	− 0.0582
Se	1.4672	− 2.7630	8.3388

**Fig. 4:**
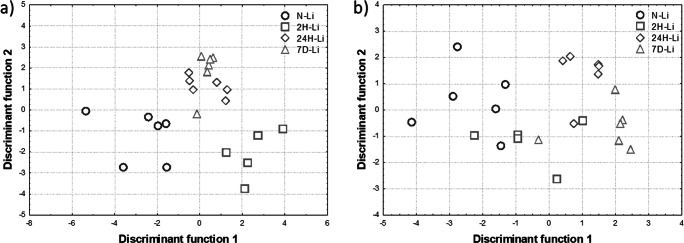
Scatterplots of observations in the space of discriminant variables obtained for the liver when all the primary variables (**a**) or only the elements of highest significance for the model—Ca, Cu and Zn (**b**)—were taken into account in analysis

Based on the data included in Table 9, three discriminant functions could characterize the object instead of eight original variables. Equations (6) and (7) define how the first two discriminant functions depend on raw primary variables. These equations were used to obtain the scatterplot of observations in the space of discriminant functions which is presented in Fig. 4a.

6 $$ {D}_1=-11.0960+0.0017P+0.0059S\hbox{--} 0.0083 Ca+0.0129 Fe-3.5453 Cu+0.1453 Zn+1.4672 Se $$

7 $$ {D}_2=18.0359-0.0008P-0.0026S\hbox{--} 0.0005K+0.0070 Ca-0.0032 Fe+0.6294 Cu-0.1160 Zn-2.7630 Se $$

Figure 4a shows that livers taken from normal and 2H rats were well separated in the space of discriminant variables from those taken from two remaining experimental groups. Thus, standard discriminant analysis based on concentrations of elements in livers allowed the differentiation of IONPs-treated rats from controls.

In Table 10, the parameters used to determine the elements of the highest significance for the model in the case of liver were presented. They indicate Ca, Cu, and Zn as the elements most important for distinguishing the livers taken from IONPs-treated animals. Therefore, further analysis was conducted only for these variables and the new raw canonical coefficients of discriminant functions were presented in Table 11.

**Table 10:** Parameters describing the significance of the primary variables for the model (liver)

	Partial Wilks’ lambda	*F* Fisher’s statistics	*p* value
P	0.70	1.75	0.210
S	0.56	3.21	0.062
K	0.77	1.16	0.363
Ca	0.44	5.18	< 0.05
Fe	0.91	0.38	0.769
Cu	0.25	12.26	< 0.05
Zn	0.42	5.63	< 0.05
Se	0.83	0.82	0.509

**Table 11:** Raw canonical coefficients of discriminant functions obtained for liver when Ca, Cu, and Zn were taken into account

Element	Discriminant function		
	1	2	3
Constant value	8.9396	8.5485	− 7.1350
Ca	0.0024	0.0060	0.0068
Cu	− 1.1921	− 0.0047	1.3278
Zn	− 0.0332	− 0.1303	− 0.1008

The scatterplot of observations in the space of the first and the second discriminant functions is presented in Fig. 4b. Exclusion of the redundant variables (elements) from the model improved the separation of the cases representing the groups 24H and 7D but worsen the differentiation of the cases from the groups N and 2H.

The squared Mahalanobis distances between livers representing four examined groups together with the *F* Fisher statistics and their *p* values are included in Table 12. All the *p* values presented there are less than 5%, which confirms the statistical significance of the calculated Mahalanobis distances.

**Table 12:** Squared Mahalanobis distances obtained for liver when Ca, Cu, and Zn were taken into account

	Squared Mahalanobis dist.				*F* statistics				*p* value			
	N-Li	2H-Li	24H-Li	7D-Li	N-Li	2H-Li	24H-Li	7D-Li	N-Li	2H-Li	24H-Li	7D-Li
N-Li	0	6	13	18	-	5	12	16	-	<0.05	< 0.05	< 0.05
2H-Li	6	0	9	7	5	-	8	6	< 0.05	-	< 0.05	< 0.05
24H-Li	13	9	0	5	12	8	-	5	< 0.05	< 0.05	-	< 0.05
7D-Li	18	7	5	0	16	6	5	-	< 0.05	< 0.05	< 0.05	-

Analogically, the discriminant analysis was done based on the data concerning the elemental composition of other organs under analysis. The results obtained for kidneys are presented in Tables S1–S3 and Fig. S4 of Supplementary materials, for heart in Tables S4–S7 and Fig. S5, while for brain, spleen, and muscles in Tables S8, S9, and S10, respectively.

Due to the correlation occurring between Ca and Zn concentrations found for kidneys, it was not possible to include all the primary variables in the discriminant analysis. As the contribution of Ca in the process of group differentiation was greater than that for Zn (see the data presented in Table 13), the concentration of this element was the subject of further statistical calculations.

**Table 13:** Parameters describing the significance of Ca and Zn for the model

	Partial Wilks’ Lambda	*F* Fisher statistics
Ca	0.68	2.01
Zn	0.71	1.81

For both the kidneys and heart, three discriminant functions were distinguished but only one of them was statistically significant (Tables S1 and S4). On the basis of raw canonical coefficients of discriminant functions (Tables S2 and S5), two new discriminant variables were created for the two organs and used to present the observations in the space of discriminant functions (Figs. S4 for kidneys and S5 for heart).

For the kidneys (Fig. S4), first discriminant function gave good separation of control animals (N group) from those injected with IONPs. In turn, consideration of a second discriminant function did not contribute to a better differentiation of the examined animal groups. Table S3 shows that none of the primary variables contribute significantly to the discrimination process. Therefore, statistical analysis for the kidneys was terminated at this stage.

In the heart case, discriminant analysis based on all the primary variables allowed satisfying separation of all animal groups in the space of discriminant variables. Two elements, namely K and Se, were determined as the most relevant for the process of the distinguishing the examined populations. However, removal of the redundant variables from the model significantly worsened classification of cases compared with the analysis carried out for all primary variables what can be seen in Fig. S5. The squares of the Mahalanobis distance were, therefore, not calculated.

The analyses performed for the brain, spleen, and muscles did not allow to determine any statistically significant discriminant functions (Tables S8–S10) neither primary variables relevant for the process of group differentiation.

## Discussion and Conclusions

As can be seen from the existing literature, multivariate methods, also known as chemometrics, are frequently used in the analysis of elemental data especially in the field of life sciences [24, 33–41]. For example, hierarchical cluster analysis was successfully applied for the prediction of cancer-related Cu-binding proteins, which can serve as a source for mechanistic–molecular studies of Cu-dependent processes in cancer [42]. The method was also used for quantitative imaging of the influence of chronic Mn exposure on metal accumulation in hippocampal formation [43]. In turn, discriminant analysis was adapted inter alia, for characterization of metal profiles within serum in Alzheimer’s disease [34] and gouty arthritis [44] as well as identification of metal biomarkers of different cancers in human tissues [39, 45, 46]. In the literature, there are also many examples of the chemometric method application in the research utilizing X-ray fluorescence-based techniques [24, 36, 38, 47, 48].

Both multivariate methods, which were used in the study, proved that the elemental composition of examined organs is so unique that enables to correctly classify them into the groups that were separated during analysis or defined a priori. The shape of the dendrograms obtained for examined groups showed big similarity between animals from which organs were collected 2 h and 7 days following IONPs injection. This is in agreement with the data presented in Table 1 and also with our previous results showing that these time intervals are connected with the most intensive elemental anomalies occurring in the internal organs [20, 22]. Moreover, the discriminant analysis showed that, regardless of the animal population studied, S, Fe, Cu, and Se are the elements that play important roles in the process of organ categorization.

Unsupervised classification (cluster analysis) of cases according to the animal groups showed some regularities only for samples taken from the liver. For this organ, the rats representing different populations were usually categorized to separate clusters. Such patterns were not observed for other organs including those for which significant elemental abnormalities occurred as a result of exposure to NPs. Better results were obtained using discriminant analysis. The scatterplots of observations were obtained for the liver, kidneys, and heart. For other examined tissues, non-statistically significant discriminant functions were determined. For the three mentioned above organs, our previous investigations showed significant elemental anomalies in NPs-treated rats [20, 22] compared with controls, and such a result is probably connected with the functions performed by these organs. The discriminant analysis carried out for the liver, kidneys, and heart using all primary variables allowed good distinguishing of the control group from IONPs-treated animals. Therefore, information on elemental composition of one of the three mentioned organs supported by this multivariate method could be used for verification whether the animals were or were not treated with NPs.

Based on the partial Wilks lambdas, the elements of highest significance in the process of group discrimination were found. In the case of liver, these were Cu, Ca, and Zn. As one can notice from Table 1, the level of Cu decreased while those of Ca and Zn increased in livers of PEG-IONPs-treated rats. Regulation of the iron metabolism is one of the main liver tasks [49]. The liver is responsible for the iron storage in a form of ferritin, regulation of this element movement through the production of peptide hepcidin, and the synthesis of major iron metabolism proteins, such as transferrin and ceruloplasmin [50]. The observed temporary (in 2H group) and long-term anomalies of liver Cu level could be an effect of ceruloplasmin release to blood in response to the increased iron level and are in agreement with our earlier results demonstrating increased concentration of Cu in rat serum after PEG-IONPs administration [22].

Also, Zn and Ca concentrations were changing in the liver as a consequence of the treatment with PEG-IONPs. The levels of both elements increased between 1st and 7th day from the injection what could indicate the occurrence of inflammatory processes being a consequence of Fe-induced oxidative stress [22]. Zn together with Cu is the part of Cu,Zn-superoxide dismutase (Cu,Zn-SOD)—an oxidoreductase enzyme responsible for rapid two-step dismutation of the toxic superoxide radical to molecular oxygen and hydrogen peroxide [51]. Because of its function, Cu,Zn-SOD is a crucial component of the cellular response to oxidative stress [51]. In our study, simultaneous increase of Cu and Zn concentrations within the liver was not observed. Therefore, an elevation in the level of Cu,Zn-SOD enzyme in the organ occurring in response to iron-induced oxidative stress cannot be confirmed. On the other hand, this cannot be ruled out either, because the increase in copper level associated with Cu,Zn-SOD could be masked by the release of copper, probably bound to ceruloplasmin, from the liver which was observed within our previous study and was described in details elsewhere [20, 22].

Significant increase of Ca content, observed for the liver between 1st and 7th day after PEG-IONPs administration could point at the role of Ca in signaling of increased production of reactive oxygen species [52]. In turn, the disturbances in the calcium economy resulting from persistent oxidative stress could lead to the loss of efficiency of adaptive mechanisms and, consequently, to the appearance of apoptosis [53].

Kidneys were another organ for which discriminant analysis allowed to distinguish the control group from IONPs-treated rats. However, in contrary to liver, the determination of elemental markers of NPs exposure was not possible for them. Kidneys are responsible for removal of small unnecessary or toxic particles from the organism [54]. However, this ability is strictly connected with the size of these elements. It was shown that nanoparticles with the diameter higher than 10 nm cannot be removed from the organism by the kidneys [21, 55]. Our previous results are in agreement with these data showing the redistribution process of PEG-IONPs [20].

In the case of heart, the discriminant analysis allowed both to differentiate normal rats from those injected with NPs and to determine the elements significant for the discrimination process, namely K and Se. Despite the lack of statistically significant changes in the iron level occurring in hearts of animals subjected to PEG-IONPs, the intense flow of blood with NPs through the organ may trigger long-term anomalies manifesting, among others, in elemental changes of the organ [20, 56]. In the recent literature, there is no unambiguous information about the nature of the observed changes. Some authors indicate a negative effect on heart related to exposure to IONPs [57], while others show their protective nature [58, 59]. Therefore, further research in this area should be carried out.

Potassium is an element of great importance for proper functioning of the nervous and muscular systems with particular emphasis on myocardial work. The right level of potassium lowers blood pressure, has positive effect on the endothelium of the vessels, and also reduces the production of free radicals what decrease the risk of brain stroke [59, 60]. What is more, the extracellular potassium concentration influences the electrical stability of the heart [60]. Increases of extracellular potassium ions concentration in serum are responsible for many early electrophysiological changes, and as a result can result in cardiac arrhythmias in both physiological and ischemic myocardium [60]. Therefore, determination of K as the element important for differentiation of normal rats from those treated with NPs could indicate some pathological processes occurring in heart. Such a conclusion is in agreement with our previous observation, showing increased Ca levels in the organ of IONPs-treated animals [20]. Ca plays an important role in the regulation of secretion and release of hormones into the bloodstream. Moreover, it influences the transmission of nerve impulses and muscle contractility [61]. Also, proper functioning of the vascular system and myocardium as well as coagulation process are associated with the occurrence of calcium ions [62, 63].

The second element proposed as the biomarker of PEG-IONPs action on heart was Se. The role of selenium in living organism is mainly connected with regulation of the inflammatory response, antioxidant properties, the proliferation/differentiation of immune cells, and optimal functioning of the cardiovascular system [64]. Selenium in a form of selenoproteins regulates various signaling processes by influencing the redox homeostasis and cellular Ca^2+^ influx [65, 66]. The determination of Se as an element important for hearts discrimination together with the fact that the level of Ca was disturbed in the organ seem to confirm the existence of oxidative stress in rats treated with PEG-IONPs.

Reassuming the obtained results, it could be concluded that the applied chemometric techniques enabled correct classification of tissues according to the organ of origin and, what is more significant, discrimination of rats subjected to NPs action from normal animals. Additionally, in the case of liver and heart, it was possible to determine the elements of the highest significance for the differentiation process which may be the candidates for the markers of exposure to nanomaterials which could potentially be applied in diagnostic procedures or medical treatments.

This study should be continued. Especially, additional technique providing the direct evidence for the deposition of PEG-IONPs (e.g., TEM) in the organs should be used. Neither TEM analysis nor histological staining was used in this study, as both techniques require appropriate and different protocols of biological material preparation. While, the reliable elemental studies are possible only using unfixed tissues. The tissue which, after a short perfusion with saline, was not fixed but only frozen, cannot be the subject of immunolabeling. Also for TEM, a special fixative (glutaraldehyde with para-formaldehyde) must be used. However, as we have shown it in our previous paper, the fixation may strongly affect the elemental composition of the tissue [67]. Therefore, we decided to carry out the elemental analysis on unfixed samples what, unfortunately, strongly limited the possibilities of their further examinations.

On the other hand, both histological and TEM study are usually limited to very low amount of the biological material. This is typically a few-micrometer-thick slice of the organ per animal. Taking into account such small amount of the sample at the beginning stage of the study, we could easily miss the areas of NPs accumulation or action. Therefore, at the first stage of the research, the general elemental anomalies occurring within organs were examined.

Our future research should also be focused on the evaluation of the blood levels of the examined elements, as such data could make easier the interpretation of the obtained statistical analysis results. Such data were not presented in this paper because of the poor optimization of the procedure of serum preparation for the analysis of Fe and other elements. The methods we used till now did not allow us to avoid the hemolysis of erythrocytes during blood centrifugation and as consequence, to obtain the reliable results of elemental analysis of serum samples.

## Electronic Supplementary Material

Supplementary Material (PDF 571 kb)

## Footnotes

1 The initial number of animals in each group was equal to 6, but because of improper injection of NP solution to one rat representing 2H group, we needed to exclude this animal from the study.
