# Supplementary material for: Organ Metallome Processed with Chemometric Methods Enable the Determination of Elements that May Serve as Markers of Exposure to Iron Oxide Nanoparticles in Male Rats
Source: Biol Trace Elem Res. 2020 Mar 12;198(2):602–16. doi: 10.1007/s12011-020-02104-z (PMC7561579; doi:10.1007/s12011-020-02104-z)
Supplement: Supplementary file 1 — (PDF 571 kb) [file 12011_2020_2104_MOESM1_ESM.pdf]

## Supplementary materials

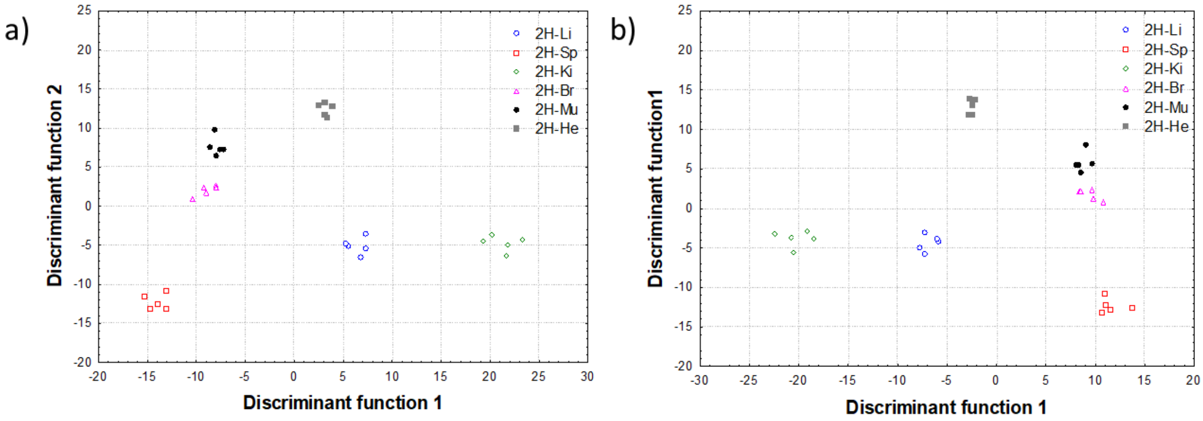


**Figure S1.** Scatterplots of observations in the space of discriminant variables obtained for 2H group when all the primary variables (a) or only elements significant for the model – S, K, Fe, Cu, Zn and Se (b) were taken into account.

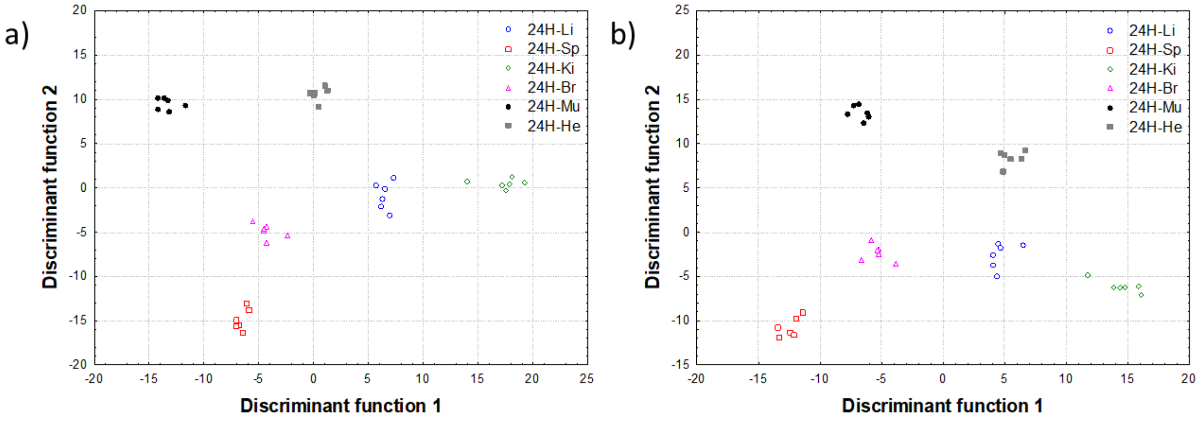


**Figure S2.** Scatterplots of observations in the space of discriminant variables obtained for 24H group when all the primary variables (a) or only elements significant for the model – P, S, Ca, Fe, Cu and Se (b) were taken into account.

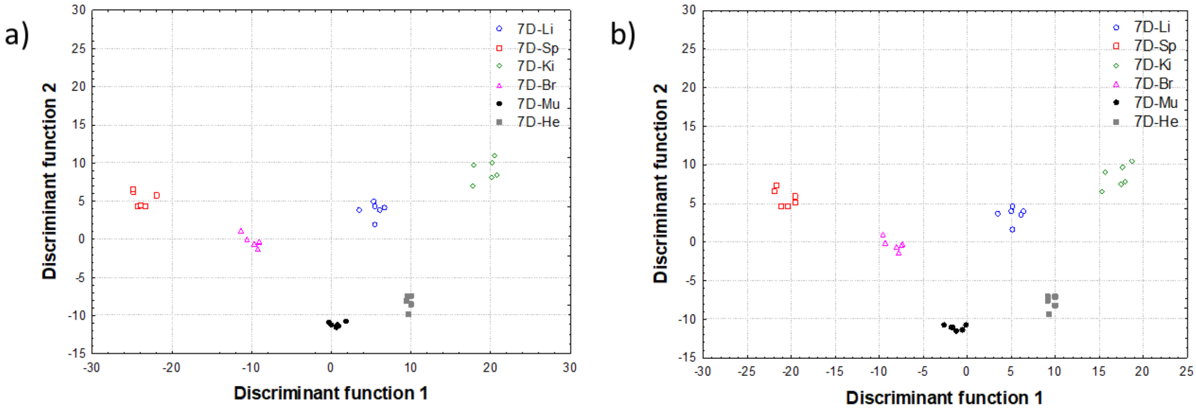


**Figure S3.** Scatterplots of observations in the space of discriminant variables obtained for 7D group when all the primary variables (a) or only elements significant for the model – P, S, K, Fe, Cu and Se (b) were taken into account.

***Kidneys***

**Table S1.** Characteristics of canonical discriminant functions determined for kidneys.

| Discriminant function | Eigenvalue | Canonical correlation | Wilks' Λ | Chi-square statistics | Df* | *p*-value** |
|---|---|---|---|---|---|---|
| **1** | 2.44 | 0.842 | 0.114 | 35.87 | 21 | <0.05 |
| **2** | 0.92 | 0.694 | 0.391 | 15.48 | 12 | 0.27 |
| **3** | 0.36 | 0.496 | 0.754 | 4.65 | 5 | 0.44 |

*Df – number of degrees of freedom for chi-square statistics;
***p*-value – observed significance level.

**Table S2.** Raw canonical coefficients of discriminant functions for kidneys.

| | **Discriminant function** | | |
|---|---|---|---|
| **Element** | **1** | **2** | **3** |
| **Constant value** | 6.8737 | 0.2610 | 3.9552 |
| **P** | 0.0038 | 0.0044 | -0.0018 |
| **S** | -0.0060 | -0.0001 | 0.0015 |
| **K** | -0.0025 | 0.0006 | 0.0009 |
| **Ca** | -0.0006 | -0.0006 | 0 |
| **Fe** | 0.0012 | 0.0211 | -0.0325 |
| **Cu** | -0.5466 | -0.4714 | 0.4737 |
| **Se** | 2.5416 | -10.4108 | -2.1847 |

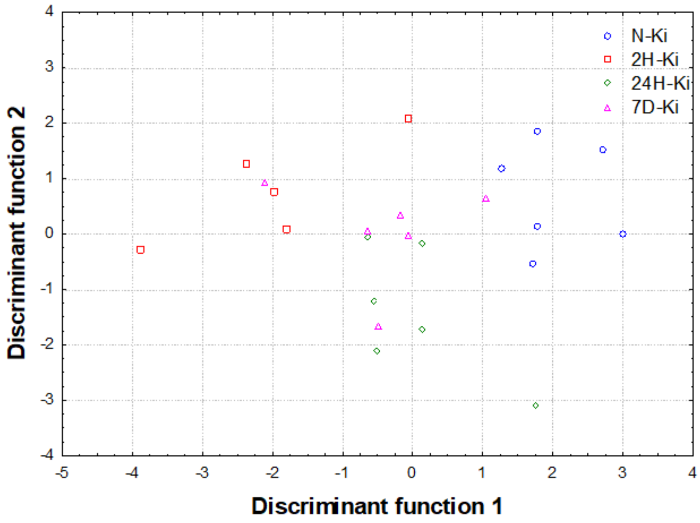


**Figure S4.** Scatterplot of observations in the space of discriminant variables obtained for kidneys when all the primary variables were taken into account in analysis.

**Table S3.** Parameters describing the significance of the primary variables for the model (kidneys).

| | Partial Wilks' lambda | F-Fisher's statistics | *p*-value |
|---|---|---|---|
| **P** | 0.73 | 1.58 | 0.24 |
| **S** | 0.67 | 2.12 | 0.14 |
| **K** | 0.77 | 1.31 | 0.31 |
| **Ca** | 0.68 | 2.01 | 0.16 |
| **Fe** | 0.72 | 1.70 | 0.21 |
| **Cu** | 0.86 | 0.70 | 0.57 |
| **Se** | 0.65 | 2.36 | 0.12 |

***Heart***

**Table S4.** Characteristics of canonical discriminant functions determined for heart.

| Discriminant function | Eigenvalue | Canonical correlation | Wilks' Λ | Chi-square statistics | Df* | *p*-value** |
|---|---|---|---|---|---|---|
| **1** | 3.95 | 0.893 | 0.070 | 39.85 | 24 | <0.05 |
| **2** | 1.22 | 0.741 | 0.348 | 15.84 | 14 | 0.323 |
| **3** | 0.30 | 0.478 | 0.771 | 3.89 | 6 | 0.692 |

*Df – number of degrees of freedom for chi-square statistics;
***p*-value – observed significance level.

**Table S5.** Raw canonical coefficients of discriminant functions for heart.

| | Discriminant function | | |
|---|---|---|---|
| **Element** | **1** | **2** | **3** |
| **Constant value** | -4.2737 | 20.1035 | 2.7906 |
| **P** | -0.0048 | -0.0028 | 0.0087 |
| **S** | 0.0094 | -0.0003 | -0.0077 |
| **K** | -0.0086 | -0.0056 | 0.0019 |
| **Ca** | 0.0026 | 0.0018 | -0.0015 |
| **Fe** | -0.0357 | 0.0604 | -0.0721 |
| **Cu** | 2.2593 | 1.1654 | -1.6437 |
| **Zn** | -0.0366 | -0.0153 | 0.0049 |
| **Se** | 40.6313 | -11.2127 | 2.9856 |

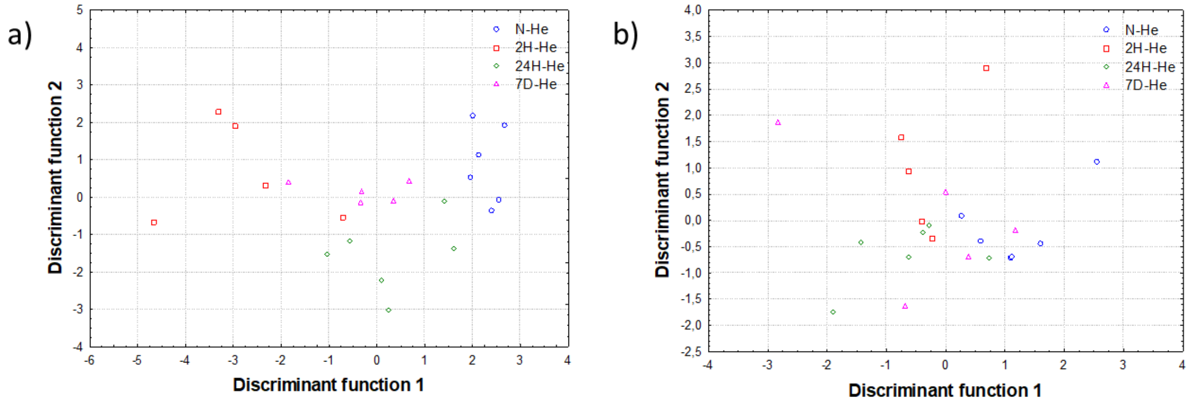


**Figure S5.** Scatterplots of observations in the space of discriminant variables obtained for the heart when all the primary variables were taken into account in analysis (a) or only the elements of highest significance for the model – K and Se (b).

**Table S6**. Parameters describing the significance of the primary variables for the model (heart).

| | **Partial Wilks' lambda** | **F-Fisher's statistics** | ***p*-value** |
|---|---|---|---|
| **P** | 0.75 | 1.20 | 0.355 |
| **S** | 0.60 | 2.48 | 0.116 |
| **K** | 0.43 | 4.86 | <0.05 |
| **Ca** | 0.53 | 3.18 | 0.067 |
| **Fe** | 0.71 | 1.51 | 0.266 |
| **Cu** | 0.82 | 0.80 | 0.520 |
| **Zn** | 0.66 | 1.92 | 0.185 |
| **Se** | 0.41 | 5.32 | <0.05 |

**Table S7.** Raw canonical coefficients of discriminant functions obtained for heart when K and Se were taken into account.

| | **Discriminant function** | |
|---|---|---|
| **Element** | **1** | **2** |
| **Constant value** | 11.1686 | 9.6114 |
| **K** | -0.0038 | -0.0006 |
| **Se** | 8.3723 | -21.9567 |

***Brain***

**Table S8.** Characteristics of canonical discriminant functions determined for brain.

| **Discriminant function** | **Eigenvalue** | **Canonical correlation** | **Wilks' Λ** | **Chi-square statistics** | **Df*** | ***p*-value**** |
|---|---|---|---|---|---|---|
| **1** | 1.86 | 0.806 | 0.16 | 29.51 | 24 | 0.20 |
| **2** | 0.67 | 0.634 | 0.45 | 12.70 | 14 | 0.55 |
| **3** | 0.32 | 0.494 | 0.75 | 4.49 | 6 | 0.61 |

*Df – number of degrees of freedom for chi-square statistics;

***p*-value – observed significance level.

***Spleen***

**Table S9.** Characteristics of canonical discriminant functions determined for spleen.

| **Discriminant function** | **Eigenvalue** | **Canonical correlation** | **Wilks' Λ** | **Chi-square statistics** | **Df*** | ***p*-value**** |
|---|---|---|---|---|---|---|
| **1** | 1.09 | 0.723 | 0.26 | 21.35 | 24 | 0.62 |
| **2** | 0.52 | 0.584 | 0.55 | 9.53 | 14 | 0.79 |
| **3** | 0.20 | 0.404 | 0.84 | 2.86 | 6 | 0.83 |

*Df – number of degrees of freedom for chi-square statistics;

***p*-value – observed significance level.

***Muscles***

**Table S10.** Characteristics of canonical discriminant functions determined for muscles.

| **Discriminant function** | **Eigenvalue** | **Canonical correlation** | **Wilks' Λ** | **Chi-square statistics** | **Df*** | ***p*-value**** |
|---|---|---|---|---|---|---|
| **1** | 1.83 | 0.804 | 0.15 | 29.95 | 24 | 0.19 |
| **2** | 0.77 | 0.659 | 0.44 | 13.30 | 14 | 0.50 |
| **3** | 0.30 | 0.479 | 0.77 | 4.17 | 6 | 0.65 |

*Df – number of degrees of freedom for chi-square statistics;

***p*-value – observed significance level.
